# Synthesis and Antiviral Properties of 1-Substituted 3-[ω-(4-Oxoquinazolin-4(3H)-yl)alkyl]uracil Derivatives

**DOI:** 10.32607/actanaturae.10983

**Published:** 2020

**Authors:** M. P. Paramonova, A. L. Khandazhinskaya, A. A. Ozerov, S. N. Kochetkov, R. Snoeck, G. Andrei, M. S. Novikov

**Affiliations:** Department of Pharmaceutical & Toxicological Chemistry, Volgograd State Medical University, Volgograd, 400131 Russia; Engelhardt Institute of Molecular Biology, Russian Academy of Science, Moscow, 119991 Russia; Rega Institute for Medical Research, KU Leuven, B-3000 Leuven, Belgium.

**Keywords:** uracil derivatives, 4-oxoquinazoline, synthesis, antiviral activity, human cytomegalovirus, varicella zoster virus

## Abstract

A series of uracil derivatives containing a 4-oxoquinazoline fragment bound to the nitrogen atom N3 of the pyrimidine ring by a short methylene bridge was synthesized to search for new antiviral agents. Some compounds in this series are shown to exhibit high inhibitory activity against human cytomegalovirus and the varicella zoster virus in a HEL cell culture.

## INTRODUCTION

Human cytomegalovirus (HCMV) is a member of the *Herpesviridae *family and belongs to the *Betaherpesvirinae *subfamily [1]. One of the key characteristics of herpes viruses, including HCMV, is their ability to induce a latent infection that can reactivate when one’s immunity is weakened [2]. Up to 90% of the adult urban population is infected with HCMV. The spectrum of diseases associated with HCMV infection ranges from a nearly asymptomatic infection to a severe multiple-organ dysfunction syndrome characterized by significant morbidity and mortality [3]. The risk group for severe HCMV infection includes transplant recipients undergoing immunosuppressive therapy [4], people with HIV infection [5], and children during the prenatal period [6]. Loss of adaptive immunity in transplant recipients and HIV-infected patients is a major risk factor for a disseminated HCMV infection, while it is assumed that immaturity of the fetal immune system predisposes infants infected *in utero *to a severe infection, congenital malformations, and stillbirths [7]. Even with the widespread use of highly active antiretroviral therapy in HIV-infected patients, HCMV is associated with a higher mortality rate not because of AIDS, but due to cerebrovascular and cardiovascular diseases [8]. In addition, studies have shown that HCMV can cause not only vascular diseases in transplant recipients [9], but also chronic inflammatory diseases such as the inflammatory bowel disease [11], accelerated immune senescence in elderly patients [11], and the development of malignant tumors [12, 13].

Ganciclovir, cidofovir, and foscarnet are the anti-HCMV drugs currently used in clinical practice to treat a HCMV infection [14]. These drugs inhibit the synthesis catalyzed by HCMV polymerase and reduce viral replication in patients presenting the clinical symptoms of an HCMV infection. However, these medicinal products cause a number of adverse effects. In particular, all of them exhibit marked toxicity [15]. In addition, these drugs are characterized by low bioavailability and need to be administered intravenously for a target blood drug concentration to be achieved. Furthermore, long-term therapy is needed for a positive outcome in the treatment of a HCMV infection; in turn, this leads to the emergence of resistant HCMV variants [16, 17, 18]. The recently approved letermovir and maribavir drugs have a significantly lower toxicity, but their prolonged use in the treatment and prevention of HCMV infections also leads to the emergence of resistant HCMV strains [19, 20]. Therefore, searching for new, highly effective anti-HCMV agents is a pressing task.

Earlier, we synthesized a series of 1-[ω-(aryloxy) alkyl]uracil derivatives containing an *N*- (4-phenoxyphenyl)acetamide fragment at the N3 nitrogen atom of the pyrimidine ring. These compounds inhibited the replication of HCMV, VZV [21], and HCV [22]. Replacing the acetamide fragment with a coumarin residue has given rise to a number of compounds that also effectively inhibit the replication of HCMV and VZV [23]. In continuation of our research focused on effective viral replication blockers, we synthesized a number of 1-[ω-(aryloxy)alkyl]uracil derivatives carrying a quinazolin-4(3H)-one moiety bound to the N3 atom in the pyrimidine ring by a linker consisting of two or three methylene groups.

## EXPERIMENTAL

All reagents were procured from Sigma and Acros Organics at the highest grade available, and they were used without further purification, unless otherwise indicated. Anhydrous DMF and isopropyl alcohol were purchased from Sigma-Aldrich Co. Anhydrous 1,2-dichloroethane and ethyl acetate were obtained by distillation over P2O5. Thin-layer chromatography (TLC) was performed on Merck TLC Silica gel 60 F254 plates by eluting with 1 : 1 ethyl acetate–hexane or a 1 : 1 ethyl acetate–1,2-dichloroethane mixture that was developed using a VL-6.LC UV lamp (Vilber). The Acros Organics (Belgium) silica gel (Kieselgur 60–200 μm, 60 A) was used for column chromatography. Yields refer to spectroscopically (1H and 13C NMR) homogeneous materials. The melting points were determined in glass capillaries on a Mel-Temp 3.0 apparatus (Laboratory Devices Inc., USA). The NMR spectra were recorded using Bruker Avance 400 (400 MHz for 1H and 100 MHz for 13C) and Bruker Avance 600 (600 MHz for 1H and 150 MHz for 13C) spectrometers in DMSO*-d*6 or CDCl3 with tetramethylsilane used as an internal standard.

The starting 3-(ω-bromoalkyl)quinazolin-4(3*H*)-one derivatives **4–7 **were obtained in accordance with the previously described methods [24].

**General procedure for synthesizing 3-(ω-bromoalkyl) quinazolin-4(3H)-one derivatives 4–7**

A mixture of quinazolin-4(3*H*)-one **1–3 **(27.37 mmol), 1,2-dibromoethane or 1,3-dibromopropane (0.116 mmol), and K_2_CO_3_ (5.0 g, 36.18 mmol) was stirred in a DMF solution (80 mL) at 70°C for 36 h. The reaction mass was evaporated to dryness in vacuo; the residue was treated with water (100 mL); the solid residue was filtered off, dried at room temperature, purified by flash chromatography eluting with ethyl acetate; and the fractions containing the product were combined and evaporated under reduced pressure. The residue was recrystallized from a 1 : 2 ethyl acetate–hexane mixture.

*3-(2-Bromoethyl)quinazolin-4(3H)-one (4). *Yield, 58%; mp, 109.5–111°C; R*f*, 0.26 (ethyl acetate–hexane, 1:1). 1H NMR spectrum (DMSO-D6) δ, ppm, *J *(Hz): 3.86 (2H, t, *J *= 6.3, BrCH_2_), 4.40 (2H, t, *J *= 6.3, NCH_2_), 7.55 (1H, dt, *J *= 7.2 and 1.1, H-5), 7.69 (1H, d, *J *= 8.1, H-8), 7.84 (1H, dt, *J *= 8.6 and 1.6, H-7), 8.17 (1H, dd, *J *= 9.0 and 1.1, H-6), 8.43 (1H, s, H-2). 13C NMR spectrum (DMSO-D6) δ, ppm: 31.1, 47.9, 121.8, 126.5, 127.5, 127.7, 135.0, 148.1, 148.4, 160.6.

*3-(3-Bromopropyl)quinazolin-4(3H)-one (5). *Yield, 59%; mp, 111–112.5°C; R*f*, 0.22 (ethyl acetate–hexane, 1:1). 1H NMR spectrum (DMSO-D6) δ, ppm, *J *(Hz): 2.27 (2H, q, *J *= 6.8, CH_2_), 3.57 (2H, t, *J *= 6.5, BrCH_2_), 4.09 (2H, t, *J *= 7.0, NCH_2_), 7.53 (1H, dt, *J *= 7.0 and 1.0, H-5), 7.66 (1H, d, *J *= 8.1, H-8), 7.81 (1H, dt, *J *= 7.0 and 1.4, H-7), 8.15 (1H, dd, *J *= 7.9 and 1.2, H-6), 8.35 (1H, s, H-2). 13C NMR spectrum (DMSO-D6) δ, ppm: 31.4, 45.0, 121.6, 126.0, 126.9, 127.1, 134.2, 147.9, 160.2.

*3-(2-Bromoethyl)-6-methylquinazolin-4(3H)-one (6). *Yield, 52%; mp, 157.5–159°C; R*f*, 0.27 (ethyl acetate– hexane, 1:1). 1H NMR spectrum (DMSO-D6) δ, ppm, *J *(Hz): 2.44 (3H, s, CH_3_), 3.85 (2H, t, *J *= 6.3, BrCH_2_), 4.39 (2H, t, *J *= 6.2, NCH_2_), 7.58 (1H, d, *J *= 8.3, H-7), 7.65 (1H, dd, *J *= 8.4 and 2.0, H-8), 7.95 (1H, t, *J *= 0.8, H-5), 8.37 (1H, s, H-2). 13C NMR spectrum (DMSO-D6) δ, ppm: 21.3, 31.1, 40.6, 47.9, 125.9, 127.3, 136.2, 136.3, 137.5, 146.0, 147.7.

*3-(2-Bromethyl)-7-chloroquinazolin-4(3H)-one (7). *Yield, 63%; mp, 138.5–140°C; R*f*, 0.41 (ethyl acetate– hexane, 1:1). 1H NMR spectrum (DMSO-D6) δ, ppm, *J *(Hz): 3.81 (2H, t, *J *= 6.3, BrCH_2_), 4.36 (2H, t, *J *= 6.2, NCH_2_), 7.56 (1H, dd, *J *= 8.5 and 1.9, H-5), 7.72 (1H, d, *J *= 1.7, H-8), 8.13 (1H, d, *J *= 8.6, H-6), 8.41 (1H, s, H-2). 13C NMR spectrum (DMSO-D6) δ, ppm: 30.5, 47.4, 120.2, 126.4, 127.4, 128.1, 139.2, 148.9, 149.3, 159.6.

**General procedure for synthesizing**

A suspension of 1-[ω-(4-bromophenoxy) alkyl]uracil derivative **8 **(1.538 mmol) and K_2_CO_3_ (0.3 g, 2.171 mmol) was stirred in a DMF solution (10 mL) at 80°C for 1 h; bromide **4–7 **(1.541 mmol) was added, and the resulting mixture was stirred at the same temperature for 24 h. The reaction mass was evaporated in vacuo; the residue was treated with water (100 mL); the solid residue was filtered off, dried at room temperature, and purified by flash chromatography on silica gel eluting with ethyl acetate; the fractions containing the product were combined and evaporated under reduced pressure; the residue was recrystallized from a 1 : 1 ethyl acetate–1,2-dichloroethane mixture.

*1-[3-(4-Bromophenoxy)propyl]-3-[2-(4-oxoquinazolin- 4(3H)-yl)ethyl]uracil (9). *Yield, 78%; mp, 178.5–179.5°C; R*f*, 0.45 (1,2-dichloroethane–MeOH, 10:1). 1H NMR spectrum (DMSO-D6) δ, ppm, *J *(Hz): 1.82 (2H, q, *J *= 6.3, CH_2_), 3.72 (2H, t, *J *= 6.6, N1CH_2_), 3.86 (2H, t, *J *= 6.2, OCH_2_), 4.15–4.20 (4H, m, CH_2_ × 2), 5.52 (1H, d, *J *= 7.8, H5), 6.83 (2H, d, *J *= 9.1, H-3’, H-5’), 7.40 (2H, d, *J *= 9.0, H-2’, H-6’), 7.45 (1H, dt, *J *= 7.6 and 1.0, H-5”), 7.54 (1H, d, *J *= 7.9, H6), 7.58 (1H, d, *J *= 8.1, H-8”), 7.74 (1H, dt, *J *= 7.7 and 1.5, H-7”), 8.03 (1H, dd, *J *= 8.0 and 1.2, H-6”), 8.18 (1H, s, H-2”). 13C NMR spectrum (DMSO-D6) δ, ppm: 27.8, 44.4, 46.9, 65.5, 100.4, 112.5, 117.3, 121.9, 126.5, 127.3, 127.6, 132.6, 134.6, 145.0, 148.2, 151.6, 158.1, 161.1, 163.0.

*1-[4-(4-Bromophenoxy)butyl]-3-[2-(4-oxoquinazolin- 4(3H)-yl)ethyl]uracil (10). *Yield, 76%; mp, 191–192°C; R*f*, 0.45 (1,2-dichloroethane–MeOH, 10:1). 1H NMR spectrum (DMSO-D6) δ, ppm, *J *(Hz): 1.47–1.56 (4H, m, CH_2_ × 2), 3.59 (2H, t, *J *= 6.3, N1CH_2_), 3.81 (2H, t, *J *= 6.0, OCH_2_), 4.17–4.22 (4H, m, CH_2_ × 2), 5.57 (1H, d, *J *= 7.9, H5), 6.84 (2H, d, *J *= 9.0, H-3’, H-5’), 7.40 (2H, d, *J *= 9.0, H-2’, H-6’), 7.42 (1H, dt, *J *= 7.2 and 1.2, H-5”), 7.56 (1H, d, *J *= 8.1, H-8”), 7.61 (1H, d, *J *= 7.9, H6), 7.71 (1H, dt, *J *= 7.7 and 1.5, H-7”), 8.04 (1H, dd, *J *= 8.0 and 1.1, H-6”), 8.17 (1H, s, H-2”). 13C NMR spectrum (DMSO-D6) δ, ppm: 25.2, 25.8, 44.4, 48.9, 67.8, 100.4, 112.3, 117.2, 121.9, 126.5, 127.2, 127.5, 132.6, 134.5, 144.9, 148.2, 148.3, 151.7, 158.3, 161.1, 162.9.

*1-[5-(4-Bromophenoxy)pentyl]-3-[2-(4-oxoquinazolin- 4(3H)-yl)ethyl]uracil (11). *Yield, 73%; mp, 174.5–176°C; R*f*, 0.47 (1,2-dichloroethane-MeOH, 10:1). 1H NMR spectrum (DMSO-D6) δ, ppm, *J *(Hz): 1.23 (2H, q, *J *= 5.6, CH_2_), 1.38 (2H, q, *J *= 7.0, CH_2_), 1.58 (2H, q, *J *= 7.3, CH_2_), 3.54 (2H, t, *J *= 7.1, N1CH_2_), 3.86 (2H, t, *J *= 6.2, OCH_2_), 4.16–4.20 (4H, m, CH_2_ × 2), 5.55 (1H, d, *J *= 7.9, H5), 6.85 (2H, d, *J *= 9.0, H-3’, H-5’), 7.39 (2H, d, *J *= 9.0, H-2’, H-6’), 7.44 (1H, dt, *J *= 7.5 and 1.2, H-5”), 7.55–7.60 (2H, m, H6, H-8”), 7.71 (1H, dt, *J *= 7.9 and 1.6, H-7”), 8.05 (1H, ddd, *J *= 7.9, 1.5 and 0.4, H-6”), 8.16 (1H, s, H-2”). 13C NMR spectrum (DMSO-D6) δ, ppm: 22.6, 28.2, 28.5, 44.4, 49.1, 68.0, 100.4, 112.2, 117.3, 122.0, 126.5, 127.2, 127.5, 132.6, 134.5, 144.9, 148.2, 148.4, 151.6, 158.4, 161.0, 162.3.

*1-[6-(4-Bromophenoxy)hexyl]-3-[2-(4-oxoquinazolin-4(3H)-yl)ethyl]uracil (12). *Yield, 78%; mp, 178.5– 179.5°C; R*f*, 0.48 (1,2-dichloroethane-MeOH, 10:1). 1H NMR spectrum (DMSO-D6) δ, ppm, *J *(Hz): 1.32 (2H, q, *J *= 6.5, CH_2_), 1.58–1.70 (4H, m, CH_2_ × 2), 1.94 (2H, q, *J *= 7.1, CH_2_), 3.68 (2H, t, *J *= 7.1, N1CH_2_), 3.84 (2H, t, *J *= 7.0, OCH_2_), 3.87–3.98 (4H, m, CH_2_ × 2), 5.64 (1H, d, *J *= 7.9, H5), 6.81 (2H, d, *J *= 9.0, H-3’, H-5’), 7.35 (2H, d, *J *= 9.0, H-2’, H-6’), 7.49 (1H, dt, *J *= 7.5 and 1.2, H-5”), 7.58 (1H, dd, *J *= 7.6 and 0.5, H-8”), 7.65 (1H, d, *J *= 7.9, H6), 7.78 (1H, dt, *J *= 7.8 and 1.7, H-7”), 8.05 (1H, dd, *J *= 8.0 and 1.1, H-6”), 8.37 (1H, s, H-2”). 13C NMR spectrum (DMSO-D6) δ, ppm: 22.8, 27.5, 28.5, 28.6, 38.2, 44.5, 48.9, 68.0, 100.6, 112.2, 117.2, 122.0, 126.5, 127.4, 127.6, 132.5, 134.6, 144.6, 148.4, 151.5, 158.4, 160.6, 162.9.

1-[8-(4-Bromophenoxy)octyl]-3-[2-(4-oxoquinazolin- 4(3*H*)-yl)ethyl]uracil (13). Yield, 77%; mp, 171.5–173°C; R*f*, 0.33 (ethyl acetate). 1H NMR spectrum (DMSO-D6) δ, ppm, *J *(Hz): 1.15–1.36 (10H, m, CH_2_ × 5), 1.68 (2H, q, *J *= 7.1, CH_2_), 3.54 (2H, t, *J *= 6.9, N1CH_2_), 3.94 (2H, t, *J *= 6.3, OCH_2_), 4.23 (4H, s, CH_2_ × 2), 5.60 (1H, d, *J *= 7.8, H5), 6.89 (2H, d, *J *= 8.6, H-3’, H-5’), 7.42 (2H, d, *J *= 8.6, H-2’, H-6’), 7.49 (1H, t, *J *= 7.5, H-5”), 7.61–7.64 (2H, m, H-8”, H6), 7.78 (1H, t, *J *= 7.5, H-7”), 8.09 (1H, d, *J *= 7.8, H-6”), 8.20 (1H, s, H-2”). 13C NMR spectrum (DMSO-D6) δ, ppm: 25.8, 26.0, 28.4, 29.0, 40.6, 44.4, 49.2, 68.2, 100.3, 112.2, 117.2, 121.9, 126.5, 127.2, 127.5, 132.5, 134.5, 144.9, 148.2, 151.6, 158.4, 161.0, 162.9.

*1-[10-(4-Bromophenoxy)decyl]-3-[2-(4-oxoquinazolin- 4(3H)-yl)ethyl]uracil (14). *Yield, 80%; mp, 161–162°C; R*f*, 0.38 (ethyl acetate). 1H NMR spectrum (DMSO-D6) δ, ppm, *J *(Hz): 1.15–1.40 (14H, m, CH_2_ × 7), 1.70 (2H, q, *J *= 7.3, CH_2_), 3.54 (2H, t, *J *= 7.1, N1CH_2_), 3.94 (2H, t, *J *= 6.5, OCH_2_), 4.20–4.24 (4H, m, CH_2_ × 2), 5.60 (1H, d, *J *= 7.8, H5), 6.90 (2H, d, *J *= 9.1, H-3’, H-5’), 7.43 (2H, d, *J *= 9.0, H-2’, H-6’), 7.50 (1H, t, *J *= 7.0, H-5”), 7.62 (1H, d, *J *= 7.5, H-8”), 7.64 (1H, d, *J *= 7.9, H6), 7.77 (1H, dt, *J *= 8.6 and 1.6, H-7”), 8.09 (1H, dd, *J *= 7.9 and 1.1, H-6”), 8.21 (1H, s, H-2”). 13C NMR spectrum (DMSO-D6) δ, ppm: 25.9, 26.1, 28.4, 28.96, 29.02, 29.16, 29.22, 29.3, 44.4, 49.2, 68.2, 100.3, 112.2, 117.2, 121.9, 126.5, 127.2, 127.5,132.5, 134.5, 144.9, 148.2, 148.3, 151.6, 158.4, 161.0, 163.0.

1-[12-(4-Bromophenoxy)dodecyl]-3-[2-(4-oxoquinazolin- 4(3H)-yl)ethyl]uracil (15). Yield, 73%; mp, 150–152°C; Rf, 0.39 (ethyl acetate). 1H NMR spectrum (DMSO-D6) δ, ppm, J (Hz): 1.17–1.41 (18H, m, CH_2_ × 9), 1.70 (2H, q, J = 7.6, CH_2_), 3.56 (2H, t, J = 7.3, N1CH_2_), 3.95 (2H, t, J = 6.5, OCH_2_), 4.21–4.26 (4H, m, CH_2_ × 2), 5.58 (1H, d, J = 7.9, H5), 6.89 (2H, d, J = 9.0, H-3’, H-5’), 7.41 (2H, d, J = 9.0, H-2’, H-6’), 7.49 (1H, dt, J = 7.1 and 1.1, H-5”), 7.60 (1H, d, J = 7.8, H-8”), 7.62 (1H, d, J = 7.9, H6), 7.78 (1H, dt, J = 8.5 and 1.6, H-7”), 8.11 (1H, dd, J = 7.9 and 1.2, H-6”), 8.17 (1H, s, H-2”). 13C NMR spectrum (DMSO-D6) δ, ppm: 25.9, 26.2, 28.5, 29.0, 29.15, 29.24, 29.3, 44.4, 49.2, 68.4, 100.4, 112.2, 117.3, 122.0, 126.5, 127.2, 127.5, 132.5, 134.4, 144.8, 148.1, 148.4, 151.6, 158.6, 161.0, 162.9.

1-[5-(4-Bromophenoxy)pentyl]-3-[2-(7-chloro-4-oxoquinazolin- 4(3H)-yl)ethyl]uracil (16). Yield, 82%; mp, 154–155°C; Rf, 0.59 (1,2-dichloroethane-MeOH, 10:1). 1H NMR spectrum (DMSO-D6) δ, ppm, J (Hz): 1.24 (2H, q, J = 8.0, CH_2_), 1.37 (2H, q, J = 7.5, CH_2_), 1.59 (2H, q, J = 7.6, CH_2_), 3.55 (2H, t, J = 7.3, N1CH_2_), 3.88 (2H, t, J = 6.5, OCH_2_), 4.17–4.20 (4H, m, CH_2_ × 2), 5.57 (1H, d, J = 7.8, H5), 6.87 (2H, d, J = 8.9, H-3’, H-5’), 7.40 (2H, d, J = 8.9, H-2’, H-6’), 7.49 (1H, dd, J = 8.5 and 1.9, H-5”), 7.61 (1H, d, J = 7.9, H6), 7.65 (1H, d, J = 1.8, H-8”), 8.05 (1H, d, J = 8.6, H-6), 8.25 (1H, s, H-2”). 13C NMR spectrum (DMSO-D6) δ, ppm: 22.1, 27.7, 28.1, 44.1, 48.6, 67.5, 99.8, 111.7, 116.7, 120.2, 126.2, 127.1, 128.1, 132.1, 138.8, 144.5, 148.9, 149.2, 151.1, 157.9, 160.0, 162.4.

1-[5-(4-Bromophenoxy)pentyl]-3-[3-(4-oxoquinazolin- 4(3H)-yl)propyl]uracil (17). Yield, 87%; mp, 103.5– 104.5°C; Rf, 0.48 (1,2-dichloroethane-MeOH, 10:1). 1H NMR spectrum (DMSO-D6) δ, ppm: 1.31 (2H, q, J = 5.6, CH_2_), 1.36–1.70 (4H, m, CH_2_ × 2), 1.94 (2H, q, J = 7.0, CH_2_), 3.68 (2H, t, J = 7.1, N1CH_2_), 3.81–3.91 (4H, m, CH_2_ × 2), 3.95 (2H, t, J = 7.3, OCH_2_), 5.64 (1H, d, J = 7.9, H5), 6.81 (2H, d, J = 9.0, H-3’, H-5’), 7.34 (2H, d, J = 9.0, H-2’, H-6’), 7.48 (1H, dt, J = 7.5 and 1.1, H-5”), 7.61 (1H, dd, J = 7.6 and 0.5, H-8”), 7.64 (1H, d, J = 7.9, H6), 7.77 (1H, dt, J = 7.7 and 1.6, H-7”), 8.05 (1H, dd, J = 8.0 and 1.1, H-6”), 8.37 (1H, s, H-2”). 13C NMR spectrum (DMSO-D6) δ, ppm: 22.8, 27.5, 28.5, 28.6,38.3, 44.5, 49.0, 68.0, 100.6, 112.2, 117.2, 122.0, 126.5, 127.4, 127.6, 132.5, 134.6, 144.6, 148.4, 151.5, 158.4, 160.6, 162.9.

1-[5-(4-Bromophenoxy)pentyl]-3-[2-(6-methyl-4-oxoquinazolin- 4(3H)-yl)ethyl]uracil (18). Yield, 79%; mp 180–181.5°C; Rf, 0.29 (ethyl acetate). 1H NMR spectrum (DMSO-D6) δ, ppm: 1.28 (2H, q, J = 6.5, CH_2_), 1.39 (2H, q, J = 6.8, CH_2_), 1.60 (2H, q, J = 7.2, CH_2_), 2.40 (3H, s, CH_3_), 3.58 (2H, t, J = 7.1, N1CH_2_), 3.89 (2H, t, J = 6.4, OCH_2_), 4.21 (4H, m, CH_2_ × 2), 5.61 (1H, d, J = 7.8, H5), 6.89 (2H, d, J = 9.1, H-3’, H-5’), 7.44 (2H, d, J = 9.0, H-2’, H-6’), 7.51 (1H, d, J = 8.3, H-7”),7.58 (1H, dd, J = 8.3 and 1.9, H-8”),7.64 (1H, d, J = 7.9, H6), 7.88 (1H, s, H-5”), 8.15 (1H, s, H-2”). 13C NMR spectrum (DMSO-D6) δ, ppm: 21.2, 22.6, 28.2, 28.5, 44.3, 49.1, 67.9, 100.3, 112.2, 117.2, 121.7, 125.8, 127.4, 132.6, 135.8, 137.0, 144.9, 146.3, 147.4, 151.6, 158.4, 161.0, 162.9.

**Antiviral assays**

The compounds were evaluated against human cytomegalovirus (HCMV, strains AD-169 and Davis) and the varicella zoster virus (VZV, strains OKA and YS). The antiviral assays were based on the inhibition of virus-induced cytopathicity or plaque formation in human embryonic lung (HEL) fibroblasts. Confluent cell cultures in 96-well microplates were inoculated with 100 CCID50 of the virus (1 CCID50 being the virus dose to infect 50% of the cell culture) or 10 or 100 plaque-forming units (PFU) (for VZV and HCMV) in the presence of varied concentrations of the test compounds. Viral cytopathicity or plaque formation was recorded as soon as it reached completion in the control virus-infected cell cultures not treated with the test compounds. Antiviral activity was expressed as the EC_50_ or compound concentration required to reduce virus-induced cytopathogenicity or viral plaque formation by 50%.

**Cytostatic activity assays**

All assays were performed in 96-well microplates. A given amount of the test compound and (5–7.5) × 10^4^ tumor cells were added to each well. The cells were allowed to proliferate for 48 h (murine leukemia L1210 cells) or 72 h (human lymphocytic CEM and human cervix carcinoma HeLa cells) at 37°C in a humidified CO_2_-controlled atmosphere. At the end of the incubation period, the cells were counted using a Coulter counter. The IC_50_ (50% inhibitory concentration) was defined as the concentration of the compound that inhibited cell proliferation by 50%.

## RESULTS AND DISCUSSION

**Synthesis of the compounds**

The compounds in this series were synthesized according to *Scheme*. The starting 3-(ω-bromoalkyl)-quinazolin-4(3*H*)-one **4–7 **derivatives were obtained in accordance with the previously described method [24]. Treating quinazolin-4(3*H*)-ones **1–3 **with a 4-fold molar excess of 1,2-dibromoethane or 1,3-dibromopropane in a DMF solution in the presence of K_2_CO_3_ gave rise to the corresponding bromides **4–7 **with 52–63% yields. The 1-[ω-(4-bromophenoxy)alkyl]uracil derivatives described earlier [25] were treated with bromides **4–7 **in the DMF solution in the presence of K_2_CO_3_ to give the target 3-[ω-(4-oxoquinazolin-4(3*H*)-yl)alkyl]uracils **9–18 **with yields of 73–87%.

**Antiviral properties**

**Scheme:**
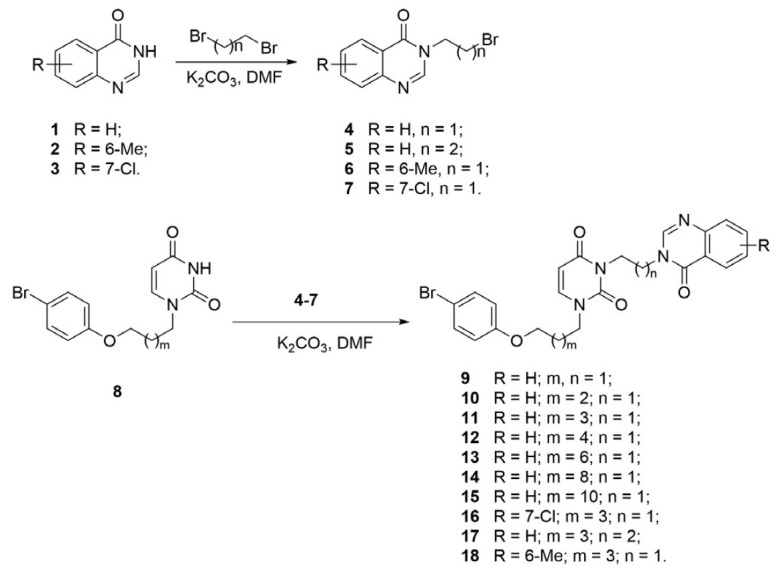


The antiviral properties of the 3-[ω-(4-oxoquinazolin-3-(4H)-yl)alkyl] derivatives of uracil **9**–**18 **against cytomegalovirus (HCMV, AD-169 and Davis strains) and the varicella zoster virus (VZV, OKA and 07-1 strains) were tested in the HEL cell culture. The results are presented in *Table*. Compound **17 **exhibited significant anti-HCMV activity: it blocked viral replication at concentrations (EC_50_) of 7.31 μM (AD-169 strain) and 5.23 μM (Davis strain). However, any structure modification, such as changing the length of the bridge m, either increasing (compounds **12–15**) or decreasing (compounds **9 **and **10**), reducing the length of the bridge n (compound **11**) or inserting substituents in the quinazoline moiety (compounds **16 **and **18**), led to a complete loss of inhibitory properties against HCMV. Compound **17 **also showed some inhibitory activity against the varicella zoster virus (VZV) and inhibited the replication of both strains of VZV at a concentration (EC_50_) of 28.96 μM. The remaining compounds were inactive (see *Table*).

**Table:** Anti-HCMV activity of 3-[ω-(4-oxoquinazolin-4(3H)-yl)alkyl]uracil derivatives 9–18 in the HEL cell culture

Compound	Antiviral activity, EC_50_/μM^a^				Cytotoxicity	
	HCMV AD-169	HCMV Davis	VZV Oka (TK^+^)	VZV 07-1 (TK^-^)	Cell morphology MCC/μM^a^	Cell growth CC_50_/μM^c^
9 (Z779)	> 100	> 100	> 100	> 100	100	-
10 (Z780)	> 20	> 100	> 20	> 100	20	-
11 (Z785)	> 20	> 20	> 20	> 100	100	-
12 (Z786)	> 100	> 20	> 100	> 100	100	-
13 (Z796)	100 >	100	> 100	> 100	> 100	12.8
14 (Z797)	> 100	> 100	> 100	> 100	> 100	> 100
15 (Z798)	> 100	> 100	> 100	> 100	≥ 100	> 100
16 (Z770)	> 20	> 20	> 20	> 100	20	-
17 (Z696)	7.31	5.23	28.96	28.96	20	1.81
18 (Z799)	> 100	> 100	> 100	> 100	> 100	> 100
Ganciclovir	2.4	2.01	-	-	350	196.41
Cidofovir	0.38	0.38	-	-	300	129.43
Acyclovir	-	-	1.6	30.37	> 440	> 100
Brivudine	-	-	0.039	6.04	> 300	> 100

^a^Effective concentration required to reduce virus plaque formation by 50%;

^b^Minimum cytotoxic concentration that causes a microscopically detectable alteration of cell morphology;

^c^Cytotoxic concentration required to reduce cell growth by 50%.

## CONCLUSIONS

Thus, we have discovered an efficient inhibitor of HCMV and VZV replication in a cell culture which contains a 4-oxoquinazoline moiety linked to the uracil residue by a chain consisting of three methylene groups. Compound **17 **can be a platform to perform targeted searches of anti-HCMV drugs.

## Abbreviations

HCMV: human cytomegalovirus
HIV: human immunodeficiency virus
AIDS: acquired immunodeficiency syndrome
VZV: varicella zoster virus
DMF: dimethylformamide
